# Quantification of Size-Binned Particulate Matter in Electronic Cigarette Aerosols Using Multi-Spectral Optical Sensing and Machine Learning

**DOI:** 10.3390/s24217082

**Published:** 2024-11-03

**Authors:** Hao Jiang, Keith Kolaczyk

**Affiliations:** Department of Biomedical Engineering, Lawrence Technological University, 21000 W 10 Mile Road, Southfield, MI 48075, USA; kkolaczyk@ltu.edu

**Keywords:** vaping, neural network, aerosol, e-liquid, atomizer, particulate matter, optical attenuation, inhalation pressure, puff topography, lung deposition

## Abstract

To monitor health risks associated with vaping, we introduce a multi-spectral optical sensor powered by machine learning for real-time characterization of electronic cigarette aerosols. The sensor can accurately measure the mass of particulate matter (PM) in specific particle size channels, providing essential information for estimating lung deposition of vaping aerosols. For the sensor’s input, wavelength-specific optical attenuation signals are acquired for three separate wavelengths in the ultraviolet, red, and near-infrared range, and the inhalation pressure is collected from a pressure sensor. The sensor’s outputs are PM mass in three size bins, specified as 100–300 nm, 300–600 nm, and 600–1000 nm. Reference measurements of electronic cigarette aerosols, obtained using a custom vaping machine and a scanning mobility particle sizer, provided the ground truth for size-binned PM mass. A lightweight two-layer feedforward neural network was trained using datasets acquired from a wide range of puffing conditions. The performance of the neural network was tested using unseen data collected using new combinations of puffing conditions. The model-predicted values matched closely with the ground truth, and the accuracy reached 81–87% for PM mass in three size bins. Given the sensor’s straightforward optical configuration and the direct collection of signals from undiluted vaping aerosols, the achieved accuracy is notably significant and sufficiently reliable for point-of-interest sensing of vaping aerosols. To the best of our knowledge, this work represents the first instance where machine learning has been applied to directly characterize high-concentration undiluted electronic cigarette aerosols. Our sensor holds great promise in tracking electronic cigarette users’ puff topography with quantification of size-binned PM mass, to support long-term personalized health and wellness.

## 1. Introduction

Health risks associated with the use of electronic cigarettes (e-cigarettes) have become an important research topic in recent years, as the rising popularity of vaping, especially among younger populations, has followed an alarming trend [1,2,3,4,5,6,7,8,9,10,11,12,13,14,15,16]. To understand the negative health consequences of vaping, it is essential to characterize e-cigarette aerosols and users’ vaping patterns through quantitative measures. Chemical analysis of e-cigarette aerosols focuses on quantification of addictive, toxic, and carcinogenic chemicals in e-cigarette aerosols, including nicotine, aldehydes, acrolein, diacetyl, and heavy metals [3,9,10,11,12,15,17,18,19,20,21,22,23,24,25,26,27]. Physical analysis of e-cigarette aerosols mainly measures the physical properties of airborne particles, known as particulate matter (PM), in terms of particle size distribution, mass median diameter, particle number concentration, particle mass concentration, and mass of total PM, etc. [23,26,28,29,30,31,32,33]. Comprehensive protocols for testing e-cigarette aerosols have been published and continuously updated by the Cooperation Centre for Scientific Research Relative to Tobacco (CORESTA) [34]. Users’ vaping patterns are quantified by their puff topography, including puff numbers, puff frequencies, inter-puff intervals, puff durations, puff flow rates, and puff volumes [19,20,23,30,35,36,37,38,39]. Chemical and physical properties of e-cigarette aerosols are mainly determined by both hardware (e-cigarette device, e-liquid, and atomizer) and puff topography together [19,26,30,31,33,39]. Portable topography devices have been demonstrated for point-of-interest measurements of puff topography and aerosol properties [32,35,39]. In our previous work, we introduced an extension of puff topography by including the mass of total PM and demonstrated a built-in aerosol sensor that can quantify the mass of total PM and estimate the nicotine dose from a user’s every puff, independent of the user’s preference of atomizer power, puff duration, puff frequency, and inhalation pressure [40].

Optical methods for characterizing aerosols offer several advantages, including high accuracy, rapid response, a wide dynamic range, robust device construction, and a compact form factor. A laser light polarization ratio method has been applied to measure the mass median diameter of e-cigarette aerosols [32]. Wavelength-dependent mid-infrared light extinction has been demonstrated for monitoring the mass concentration of e-cigarette aerosols [41]. Photometric measurement using a laser beam has been developed to measure e-cigarette aerosol concentrations [42]. Holographic microscopy has been demonstrated for detecting the volatility of e-cigarette aerosols [43]. Angle-dependent light scattering has been implemented to measure the particle size distribution of aerosols [44,45,46]. Three laser beams of different wavelengths have been demonstrated to measure the mass concentration of aerosols [47]. In our previous works, we implemented photometric detection of scattered light to quantify the mass of total PM generated from every puff of an e-cigarette [40]. We also applied dual wavelengths of light to estimate the particle size distribution properties of e-cigarette aerosols [48,49]. Machine learning and neural networks have seen rapidly growing applications across various fields in recent years. Aerosol sensing based on light scattering and holography has been significantly enhanced by machine learning [43,45,50]. Machine learning has also been implemented to classify biological aerosols and atmospheric aerosols [51,52].

For quantifying PM in e-cigarette aerosols, in addition to the aforementioned mass of total PM in a puff, obtaining size-specific information on PM would be highly beneficial, as it enables the estimation of lung deposition of PM using the multiple-path particle dosimetry (MPPD) model [53,54,55,56]. Accurate quantification of size-binned PM mass in e-cigarette aerosols, whether through a portable device for point-of-interest monitoring or, ideally, an in-device aerosol sensor, is highly valuable for managing and studying the health risks associated with vaping. In this work, we present an aerosol sensor that can directly characterize high-concentration e-cigarette aerosols using multi-spectral optical sensing and machine learning. Using optical attenuation signals measured from three wavelengths, the masses of PM in three size bins are computed with a lightweight neural network. To the best of our knowledge, this is the first demonstration of using machine learning to directly characterize high-concentration e-cigarette aerosols. The aforementioned existing aerosol measurement techniques using machine learning are designed to characterize aerosols at low or medium concentrations or diluted e-cigarette aerosols [43,45,50,51,52].

## 2. Multi-Spectral E-Cigarette Aerosol Sensor Working Principle

**Figure 1:**
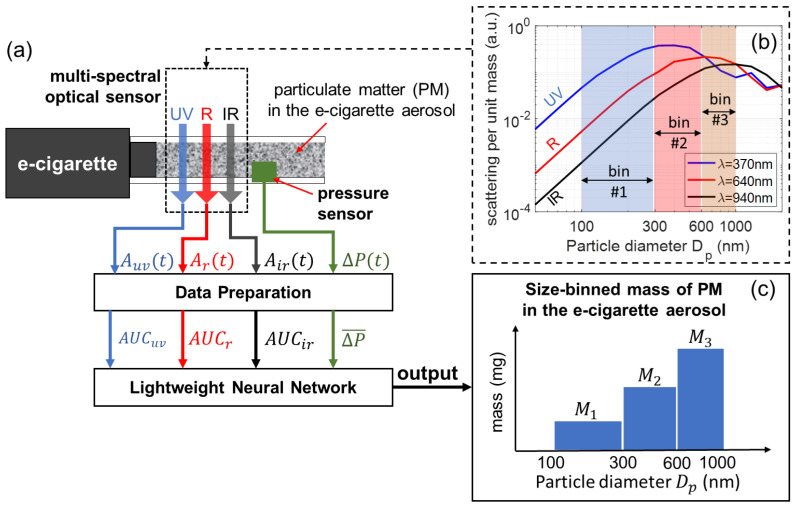
Schematic for the concept of a multi-spectral optical sensor for measuring the size-binned mass of particulate matter (PM) in e-cigarette aerosols using a neural network. (**a**) Schematic of optical configuration and the algorithm. (**b**) Scattering efficiency spectra for three wavelengths calculated based on Mie theory. (**c**) Schematic of mass of PM in three size bins.

Figure 1 shows the working principle of our multi-spectral sensor, which is connected to an e-cigarette device to measure the size-binned mass of PM in the generated vaping aerosol. Three LEDs of ultraviolet (UV), red (R), and near-infrared (IR) wavelength launch beams of light transmitting through the e-cigarette aerosol, and the transmitted intensity of each wavelength is measured and recorded in real time. A pressure sensor measures the applied air pressure that drives the aerosol flow. An e-cigarette aerosol, composed of high-concentration airborne particles (PM), is a highly scattering medium. As the light travels through the aerosol, its intensity becomes attenuated due to the absorption and scattering of light by the particles. Figure 1b shows the scattering efficiencies (scattered power per unit particle mass) as a function of particle diameter $D_{p}$, calculated for three wavelengths, 370 nm (UV), 640 nm (R), and 940 nm (IR). The calculations are based on Mie theory using an open-source software MiePlot (version 4.6) [57]. The scattering efficiency, and consequently optical attenuation, is strongly dependent on the wavelength and the size bin. It should be noted that the contrast and variation in scattering efficiencies for the three different wavelengths allows for PM of different size ranges to be differentiated. In this work, the PM of e-cigarette aerosols is studied according to three size ranges, given by three size bins marked in Figure 1b. Using optical attenuation signals from all three wavelengths, as well as the pressure signal, the mass of PM in individual size bins can be quantified using a lightweight neural network. The detailed theoretical framework for multi-spectral sensors is presented in the following.

Under the transmission mode, the optical attenuation of a beam of light for a given wavelength traveling through a medium is given by $A\left(t\right)=-log_{10}\frac{I(t)}{I_{0}}$, in which I(t) is the intensity of transmitted light through the medium as a function of time, and $I_{0}$ is the intensity of incident light. The optical attenuation for three wavelengths is given by Equation (1), in which the subscripts “uv”, “r”, and “ir” denote the terms for the ultraviolet, red, and near-infrared wavelength, respectively.

(1) $$\begin{array}{c} A_{uv}\left(t\right)=-log_{10}\frac{I_{uv}\left(t\right)}{I_{0,uv}}\\ A_{r}\left(t\right)=-log_{10}\frac{I_{r}\left(t\right)}{I_{0,r}}\\ A_{ir}\left(t\right)=-log_{10}\frac{I_{ir}\left(t\right)}{I_{0,ir}} \end{array}$$

Optical attenuation through a highly scattering medium, such as an e-cigarette aerosol, can be precisely described by the modified Beer–Lambert law, given in Equation (2) [58,59,60,61,62]. Considering a monodispersed aerosol with a concentration of *c*, the optical attenuation for wavelength λ caused by this medium can be given by

(2) $$A_{\lambda }=\epsilon_{\lambda }\cdot c\cdot L\cdot DPF_{\lambda }+G_{\lambda }$$

in which $\epsilon_{\lambda }$ is the extinction coefficient (including absorption and scattering) of the particle for wavelength λ, *L* is the distance between the light source and the detector, $DPF_{\lambda }$ is the differential path length factor to account for the increase in optical path length for scattered light, and $G_{\lambda }$ is a geometry-related factor [58,60]. The optical attenuation depends on both wavelength and particle size and scales up with increasing PM concentration.

As illustrated in Figure 1, the total PM mass for the three size bins are given by $M_{1}$, $M_{2}$, and $M_{3}$, for size bin #1 (100–300 nm), size bin #2 (300–600 nm), and size bin #3 (600–1000 nm), respectively. Considering the dynamic process of an e-cigarette aerosol flowing across the sensor, the instantaneous size-binned PM mass concentration (mass per unit volume) of the aerosol crossing the light beam can be represented by $c_{1}\left(t\right)$, $c_{2}\left(t\right)$, and $c_{3}\left(t\right)$ for the three size bins. The instantaneous volumetric aerosol flow rate is given by Q(t). The size-binned PM mass for the three bins are given by Equation (3).

(3) $$\begin{array}{c} M_{1}=\int c_{1}\left(t\right)Q\left(t\right)dt\\ M_{3}=\int c_{2}\left(t\right)Q\left(t\right)dt\\ M_{3}=\int c_{3}\left(t\right)Q\left(t\right)dt \end{array}$$

Note that the volumetric flow rate, Q(t), is a function of the inhalation pressure ΔP(t), represented by Equation (4), if we ignore the effects of aerosol temperature and viscosity on the flow characteristics.

(4) Q(t)=U[ΔP(t)]

The optical attenuation for each wavelength can be given by a wavelength-specific function of size-binned PM mass concentration in all size channels, given in Equation (5).

(5) $$\begin{array}{c} A_{uv}\left(t\right)=F_{uv}\left[c_{1}\left(t\right),c_{2}\left(t\right),c_{3}\left(t\right)\right]\\ A_{r}\left(t\right)=F_{r}\left[c_{1}\left(t\right),c_{2}\left(t\right),c_{3}\left(t\right)\right]\\ A_{ir}\left(t\right)=F_{ir}\left[c_{1}\left(t\right),c_{2}\left(t\right),c_{3}\left(t\right)\right] \end{array}$$

It should be noted that the attenuation caused by particles smaller than 100 nm and those larger than 1000 nm has been ignored. This is an acceptable approximation considering the typical particle size distribution profile of e-cigarette aerosols, which will be further discussed in Section 4.4.

An inversion of Equation (5) will give the generalized equations relating size-binned mass concentration with the multi-spectral optical attenuation, as described in a generalized form in Equation (6).

(6) $$\begin{array}{c} c_{1}\left(t\right)=F_{1}\left[A_{uv}\left(t\right),A_{r}\left(t\right),A_{ir}\left(t\right)\right]\\ c_{2}\left(t\right)=F_{2}\left[A_{uv}\left(t\right),A_{r}\left(t\right),A_{ir}\left(t\right)\right]\\ c_{3}\left(t\right)=F_{3}\left[A_{uv}\left(t\right),A_{r}\left(t\right),A_{ir}\left(t\right)\right] \end{array}$$

By incorporating Equations (4) and (6) into Equation (3), the size-binned PM mass are given by Equation (7), which suggests that the size-binned PM mass can be calculated based on multi-spectral attenuation signals and inhalation pressure.

(7) $$\begin{array}{c} M_{1}=\int F_{1}\left[A_{uv}\left(t\right),A_{r}\left(t\right),A_{ir}\left(t\right)\right]U\left[\Delta P\left(t\right)\right]dt\\ M_{2}=\int F_{2}\left[A_{uv}\left(t\right),A_{r}\left(t\right),A_{ir}\left(t\right)\right]U\left[\Delta P\left(t\right)\right]dt\\ M_{3}=\int F_{3}\left[A_{uv}\left(t\right),A_{r}\left(t\right),A_{ir}\left(t\right)\right]U\left[\Delta P\left(t\right)\right]dt \end{array}$$

Given a known e-cigarette aerosol composition and e-cigarette aerosol flow characteristics, the functions $F_{1}$, $F_{2}$, $F_{3}$, and *U* can all be explicitly derived. More specifically, functions $F_{1}$, $F_{2}$, $F_{3}$ can be derived using Mie theory and the modified Beer–Lambert law. Function *U* can be calculated or modeled based on the aerosol fluidic channel configuration. Alternatively, machine learning algorithms can straightforwardly find the correlation between the four inputs $A_{uv}\left(t\right)$, $A_{r}\left(t\right)$, $A_{ir}\left(t\right)$, ΔP(t), and the three outputs $M_{1}$, $M_{2}$, $M_{3}$ by training the neural networks with comprehensive data.

In this work, we aim to implement a lightweight neural network to quantify the size-binned PM mass. To achieve this goal, the following assumptions are made to Equation (7) in order to simplify the neural network model. Firstly, the functions $F_{1}$, $F_{2}$, $F_{3}$ are treated as linear functions of $A_{uv}\left(t\right)$, $A_{r}\left(t\right)$, $A_{ir}\left(t\right)$ as expressed in Equation (8). In the equation, $f_{i,j}$ are coefficients specific to the particle size bin *i* and wavelength *j* (*j* = 1, 2, 3 for UV, Red, IR respectively). This simplification essentially ignores the modification of optical path length for scattered light and the geometry factor, as introduced in existing works [59,60,61,62] and reduces the modified Beer–Lambert law from Equation (2) into the generic form $A_{\lambda }=\epsilon_{\lambda }\cdot c\cdot L$ [60].

(8) $$\begin{array}{c} c_{1}\left(t\right)=F_{1}\left[A_{uv}\left(t\right),A_{r}\left(t\right),A_{ir}\left(t\right)\right]=f_{1,1}A_{uv}\left(t\right)+f_{1,2}A_{r}\left(t\right)+f_{1,3}A_{ir}\left(t\right)\\ c_{2}\left(t\right)=F_{2}\left[A_{uv}\left(t\right),A_{r}\left(t\right),A_{ir}\left(t\right)\right]=f_{2,1}A_{uv}\left(t\right)+f_{2,2}A_{r}\left(t\right)+f_{2,3}A_{ir}\left(t\right)\\ c_{3}\left(t\right)=F_{3}\left[A_{uv}\left(t\right),A_{r}\left(t\right),A_{ir}\left(t\right)\right]=f_{3,1}A_{uv}\left(t\right)+f_{3,2}A_{r}\left(t\right)+f_{3,3}A_{ir}\left(t\right) \end{array}$$

Secondly, during the brief period of time when the aerosol flow causes distinctive optical attenuation signals, the inhalation pressure can be approximated as a constant value, given as a mean inhalation pressure $\bar{\Delta P}$. Specifically, this is a good approximation if the puffs from e-cigarettes are drawn by a vaping machine, which usually applies a relatively stable pressure during the puff. Based on these approximations, Equation (7) can be reduced into Equation (9)

(9) $$\begin{array}{c} M_{1}=F_{1}\left[AUC_{uv},AUC_{r},AUC_{ir}\right]G\left[\bar{\Delta P}\right]\\ M_{2}=F_{2}\left[AUC_{uv},AUC_{r},AUC_{ir}\right]G\left[\bar{\Delta P}\right]\\ M_{3}=F_{3}\left[AUC_{uv},AUC_{r},AUC_{ir}\right]G\left[\bar{\Delta P}\right] \end{array}$$

in which the terms $AUC_{uv}$, $AUC_{r}$, $AUC_{ir}$ are the integrals of the optical attenuation for the corresponding wavelengths, given in Equation (10). “AUC” stands for “area under the curve”.

(10) $$\begin{array}{c} AUC_{uv}=\int A_{uv}\left(t\right)dt\\ AUC_{r}=\int A_{r}\left(t\right)dt\\ AUC_{ir}=\int A_{ir}\left(t\right)dt \end{array}$$

Based on Equations (9) and (10), for each puff, the PM mass $M_{1}$, $M_{2}$, $M_{3}$ in three size bins can be predicted from an input of four data: $AUC_{uv}$, $AUC_{r}$, $AUC_{ir}$, and $\bar{\Delta P}$. A lightweight neural network, as illustrated in Figure 1, is adequate for this purpose.

## 3. Materials and Methods

### 3.1. Prototype of a Multi-Spectral E-Cigarette Aerosol Sensor

**Figure 2:**
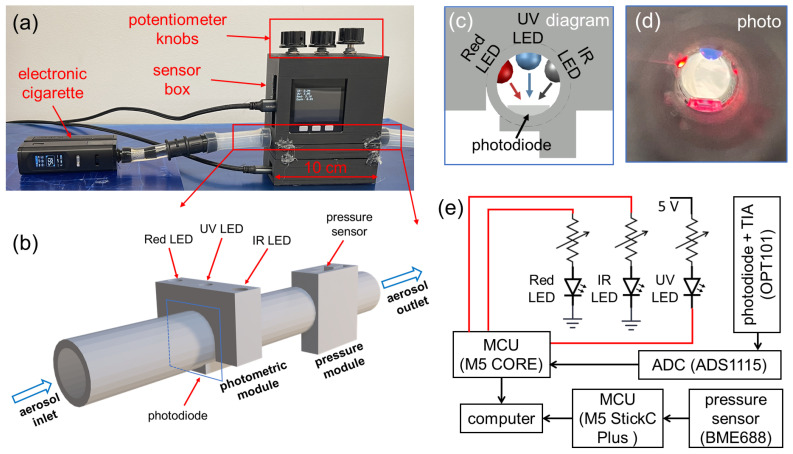
A prototype of a multi-spectral sensor for e-cigarette aerosols based on three wavelengths. (**a**) Photograph of the constructed sensor box connected to an electronic cigarette. (**b**) A 3-D schematic of the fundamental functional modules of the sensor, including a photometric module and a pressure module. (**c**) Design diagram and (**d**) photograph showing the cross-sectional view of the optical configuration inside the photometric module. (**e**) Diagram for the sensor circuit and data collection.

Figure 2a shows a multi-spectral sensor built into a sensor box connected to a commercial e-cigarette. The core functional component shown in Figure 2b includes a photometric module, a pressure module, and a cylindrical aerosol flow channel with a diameter of 10 mm. The photometric module is comprised of three LEDs in ultraviolet (UV), red (R), near-infrared (IR) wavelength, and a photodiode. Cross-sectional views of the optical configuration are given in Figure 2c,d. The three LEDs are located on the same plane perpendicular to the direction of the aerosol flow, and the transmitted light is collected by the same photodiode located on the same plane. The circuit diagram is shown in Figure 2e. The UV LED, model LED370E by Thorlabs, has a peak wavelength of 370 nm and a full-width-half-maximum (FWHM) bandwidth of 10 nm. The red LED, model LED630E by Thorlabs, has a peak wavelength of 640 nm and a bandwidth of 17 nm. The IR LED, model LED940E by Thorlabs, has a peak wavelength of 940 nm and a bandwidth of 50 nm. It should be noted that the bandwidth of the IR LED is relatively large; however, its effects on the optical signals are negligible, with an estimated relative error lower than 0.008, by comparing the overall scattering efficiency from the 50 nm band to that from a single wavelength of 940 nm. All three LEDs are connected to digital pins of a microcontroller unit (MCU), M5 CORE by M5STACK. Three separate variable resistors (potentiometers) are serially connected to the LEDs for tuning their brightness separately. Before each experiment, the three potentiometer knobs on the sensor box were adjusted to obtain strong and stable optical signals for all wavelengths, which were displayed on the M5 CORE screen.

**Figure 3:**
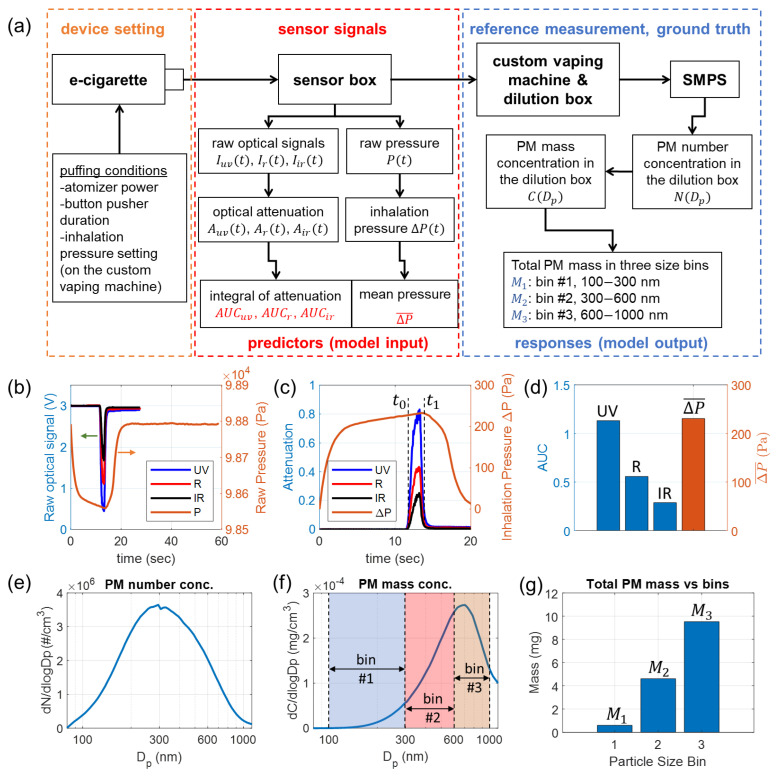
Acquisition and preparation of data for training, validation, and testing of the neural network model. The example sensor signals and reference measurement results were collected from puff No. 30 listed in Table S1. (**a**) Schematic of the experimental setup and flowcharts for sensor signal processing and reference measurement of size-binned PM mass. (**b**) Raw optical signals of the three wavelengths and raw pressure signal. (**c**) Calculated optical attenuation for three wavelengths and inhalation pressure. (**d**) Calculated integrals of optical attenuation and mean inhalation pressure, which are used as predictors (model input). (**e**) Number concentration of PM in the dilution box. (**f**) Mass concentration of PM in the dilution box. (**g**) Measured mass of total PM in three size bins, which are used as responses (model output).

The three LEDs are controlled by three digital pins with logical levels in 0 V/3.3 V (LOW/HIGH). Due to the varying forward bias voltage needed for LEDs of different wavelengths, the three LEDs are connected with digital pins in different configurations, as demonstrated in Figure 2e. The Red and IR LED are switched on by applying a digital HIGH (3.3 V), while the UV LED is switched on by a digital LOW (0 V). In the signal acquisition, the three LEDs are periodically and alternatively switched on and off, such that only one LED emits light at any given moment. Such a fashion ensures that optical signals measured by the same photodiode can be differentiated into three separate wavelengths by timing. A similar strategy has been used in the reflection-based photometric sensor in our previous work [40]. The photodiode used in this work is an optical sensor chip, OPT101 by Texas Instruments, which includes a photodiode and a transimpedance amplifier (TIA). The spectral sensitivity range of the photodiode spans 300–1100 nm, which can effectively cover all three LED wavelengths. The significant variations in spectral responsivity for three wavelengths are compensated by tuning the brightness of all three LEDs using the potentiometer knobs. The output of OPT101 is an analog signal, which is digitized by a 16-bit analog-to-digital conversion (ADC) chip, ADS1115 by Texas Instruments, into a digital signal read by the M5 CORE MCU. The sampling interval for data acquisition of each optical signal is 44 ms. In each period, the dark signal (all LEDs off), IR signal (only IR LED on), UV signal (only UV LED on), and Red signal (only Red LED on) are acquired in sequence with a 10 ms duration for each signal followed by a 1 ms gap. The dark signals, accounting for the insignificant amount of ambient light into the sensor and background noise, are subtracted from the acquired IR, UV, and Red signals. For each puff, the attained raw optical signals are given as $I_{uv}\left(t\right)$, $I_{r}\left(t\right)$, and $I_{ir}\left(t\right)$. One example of collected raw optical signals is shown in Figure 3b.

For the pressure module, a commercial gas sensor, BME688 by Bosch, is used to collect the absolute pressure. In our previous works, we have used similar pressure sensors for measuring inhalation pressure [40,48]. Another MCU, M5 StickC Plus by M5STACK, is connected to the pressure sensor to collect the readings of aerosol pressure, given as P(t). The sampling interval for the data acquisition of pressure is 338 ms. One collected pressure signal from an exemplary puff is shown in Figure 3b. Both MCUs used in the sensor were programmed using Arduino C codes.

The housing of the sensor box was fabricated using 3D printing (fused deposition modeling of PLA plastic in black color), conducted with a CREALITY K1 MAX 3D printer (Creality, Shenzhen, China). The components inserted into the photometric and pressure modules were sealed with a silicone sealant to ensure an air-tight seal. The fabricated sensor box has overall dimensions of 10 cm (width) × 12 cm (height) × 5 cm (depth).

### 3.2. E-Cigarettes and E-Liquids

Three different commercial e-cigarettes are used in this work, as shown in Figure S1. The default device used in training, validation, and testing of the neural network is an ASPIRE Nautilus PrimeX vaping mod, equipped with a Nautilus pod (atomizer coil resistance: 0.78 Ω), as shown in Figure 2a. The other two e-cigarettes are SUORIN ELITE (atomizer coil resistance: 1.0 Ω) and SMOK S-PRIV (atomizer coil resistance: 1.8 Ω), as shown in Figure S1.

Three different e-liquids are used in this work, as shown in Figure S2. The default e-liquid used in training, validation, and testing of the neural network is KSPR (manufactured by BB Vapes, Markham, Ontario, Canada), with a labeled nicotine concentration of 11.75 mg/mL. KSPR has a labeled base material comprised of 70% vegetable glycerin (VG) and 30% propylene glycol (PG), and the density of the e-liquid is 1.12 g/mL. The other two e-liquids are ENVY (manufactured by BB Vapes, labeled nicotine concentration 11.75 mg/mL) and DewBerry (manufactured by Hi-Drip E-Liquid, New York, NY, USA, labeled nicotine concentration 6 mg/mL).

### 3.3. Acquisition of Data from E-Cigarettes

Figure 3a shows a schematic for sensor signal acquisitions and reference e-cigarette aerosol measurements for collecting data for training, validation, and testing of the neural network. For each puff, the e-cigarette is activated with preset puffing conditions, and the generated aerosol travels through the sensor box. The flow is driven by the custom vaping machine. The e-cigarette device is configured with specific puffing conditions, including atomizer power, button pusher duration (to activate the atomizer), and inhalation pressure setting. The inhalation pressure is determined by the pressurization time used by the dilution box of the custom vaping machine [40]. Sensor signals, including raw optical and pressure signals, are collected for the activated puff. Further processing of the sensor signals produces the data for input (predictors) of the neural network, which is elucidated in Section 3.4. All PM in the e-cigarette aerosol generated from the puff is launched into the dilution box to be diluted and homogenized. A scanning mobility particle sizer (SMPS) measures the concentration of the diluted aerosol, which is used to calculate the size-binned PM mass for output (responses) of the neural network, to be explained in Section 3.5. A photograph of the entire experimental setup is provided in Figure S3a. It is important to clarify that our sensor directly collects signals from high-concentration, undiluted e-cigarette aerosols, whereas the reference measurements are conducted on diluted aerosols. This dilution is necessary to keep the concentration within the operational range of the SMPS system.

For training and validation of the neural network, 100 puffs were collected from the default e-cigarette (Nautilus PrimeX) and default e-liquid (KSPR) to produce a dataset of 100 trials. The experimental conditions applied on the e-cigarette were diversified to train the model for a wide range of puffing conditions: atomizer power from 15 W to 25 W, button pusher duration from 1.5 s to 3 s, and dilution box pressurization time from 7 s to 150 s. All puffing conditions for the 100 puffs are listed in Table S1. For testing the neural network, an additional 10 new puffs were collected using the puffing conditions listed in Table S2 to provide unseen data for testing the performance of the neural network. It should be noted that the puffing conditions in Table S2 implement individual parameters (atomizer power, button pusher duration, and pressurization time) of the same aforementioned ranges; however, the specific combinations of parameters applied for these 10 new puffs have not been used in Table S1. Such an experimental methodology is used to ensure the attained dataset is truly unseen by the model, but the input/output data are still within the same range as those used in training and validation.

The sensor signal processing and data preparation were all carried out using custom-programmed scripts in MATLAB (version R2024a).

### 3.4. Sensor Signal Processing

Figure 3b shows the raw optical signals $I_{uv}\left(t\right)$, $I_{r}\left(t\right)$, $I_{ir}\left(t\right)$ and pressure signals P(t) acquired from puff No. 30 listed in Table S1. The optical attenuation for three wavelengths $A_{uv}\left(t\right)$, $A_{r}\left(t\right)$, $A_{ir}\left(t\right)$ are calculated based on Equation (1). The intensity of incident light for three wavelengths $I_{0,uv}$, $I_{0,r}$, $I_{0,ir}$ are calculated from the mean of raw optical signals in a time window spanning the first 2 s (when no aerosol is present). The inhalation pressure, ΔP(t), is calculated as $\Delta P\left(t\right)=P_{amb}-P\left(t\right)$, in which $P_{amb}$ is the ambient pressure, calculated by the mean of P(t) during a time window spanning the finishing 2 s of the acquisition, in which the pressure has already returned to the ambient condition.

The calculated optical attenuation signals for three wavelengths and inhalation pressure are plotted in Figure 3c. Since the optical signals and pressure signals are sampled at different rates and timing, these signals are interpolated to the same time points. An effective time window, marked by $t_{0}$ and $t_{1}$ in Figure 3c, is first identified to cover the duration of time when the aerosol travels across the sensor. The two time stamps for the window are determined when $A_{r}\left(t\right)$ is 1/10 of the maximum attenuation. $AUC_{uv}$, $AUC_{r}$, and $AUC_{ir}$ are calculated by integration of $A_{uv}\left(t\right)$, $A_{r}\left(t\right)$, and $A_{ir}\left(t\right)$ in the time window [$t_{0}$, $t_{1}$], respectively, according to Equation (10). The mean inhalation pressure $\bar{\Delta P}$ is calculated by the mean of ΔP(t) in the time window [$t_{0}$, $t_{1}$]. The four attained values, $AUC_{uv}$, $AUC_{r}$, $AUC_{ir}$, and $\bar{\Delta P}$ are plotted in Figure 3d. They are used as input (predictors) for the neural network.

### 3.5. Reference Measurement of E-Cigarette Aerosols for the Ground Truth

In order to train the neural network and test its performance, the ground truth of the target output must be acquired through reliable approaches. In this work, we implement a custom vaping machine and an SMPS system to measure the generated e-cigarette aerosols to obtain accurate data of size-binned PM mass $M_{1}$, $M_{2}$, $M_{3}$ for every puff.

A photograph of the reference measurement system is shown in Figure S3a. The e-cigarette is loaded under a button pusher, which can automatically activate the atomizer of the e-cigarette for a preset duration. Since the concentrations of e-cigarette aerosols are too high to be directly measured by an SMPS, the generated e-cigarette aerosols must be diluted before the SMPS measurement. A custom vaping machine is used to draw the flow of the generated e-cigarette aerosol into a dilution box, which can dilute the aerosol by a factor from 300 to 1000, depending on the volume of the e-cigarette aerosol. The system’s functioning diagram and flowchart of operation are plotted in Figure S3b,c. A similar experimental setup based on the same custom vaping machine was used in our previous work for testing a puff topography sensor [40].

Before acquisition, the dilution box is cleaned and filled with clean air (i.e., no PM in the box). When an acquisition sequence is activated, valves MBV1 and SV2 open, and pump P2 turns on to pump the air out of the dilution box for a given amount of pressurization time to create a negative pressure in the dilution box. This negative pressure serves as the inhalation pressure, and it increases with a longer pressurization time. Near the end of the pressurization, the button pusher (BP) is activated to push the firing button of the e-cigarette for the preset duration. The generated e-cigarette aerosol is first measured by the sensor box and then enters the dilution box through MBV1. After all of the aerosol has been captured inside the dilution box, MBV1 and SV2 close. Valve SV1 opens and pump P1 turns on to pump air into the box for 10 s in order to quickly stabilize the pressure inside the dilution box. The aerosol is mixed with clean air in the box to become homogenized for 20 s. After dilution and homogenization, SV1 and MBV2 open, and the diluted aerosol enters the SMPS system, which measures the particle number concentration of the diluted aerosol, given as $N(D_{p})$. After the SMPS measurement, all valves close, and the box is opened to remove all PM in the box. During the acquisition sequence, all valves and pumps are controlled through relays connected to an MCU, Arduino Mega2560, which was programmed using Arduino C.

The SMPS system plays an essential role in this reference e-cigarette aerosol measurement. The SMPS system, manufactured by TSI, as shown in Figure S3a, is comprised of an electrostatic classifier (EC), a differential mobility analyzer (DMA), a soft X-ray neutralizer, and a condensation particle counter (CPC). The inlet aerosol flow rate for EC is 0.4 L/min, and the CPC inlet flow rate is 1 L/min. A custom-made flow equalizer is used at the CPC inlet to split the flow. For the sheath flow in the DMA column, a relatively low sheath flow rate, 2 L/min, is used in our measurements, as it allows the system to measure PM with diameters up to 1170 nm. The decided range for scanning $D_{p}$ is from 60 nm to 1170 nm, which can effectively cover all three size bins studied in this work. The measurement result from SMPS is the particle number concentration $N(D_{p})$, which describes the number of particles in the size channel centered at $D_{p}$ per unit volume (i.e., per cm^3^ volume of the diluted aerosol). In this work, the SMPS system scans $D_{p}$ with 64 channels per decade resolution. To clearly display the particle size dependence over a wide range, the value $\frac{dN}{dlogD_{p}}$ is usually used in aerosol measurement results, as plotted in Figure 3e, which represents the differential number concentration per logarithmic interval of $D_{p}$. Based on the acquired particle number concentration $N(D_{p})$, the PM mass concentration $C(D_{p})$ can be directly calculated in Equation (11), assuming all particles are in spherical shapes.

(11) $$\begin{array}{c} C\left(D_{p}\right)=\frac{4}{3}\pi {\left(\frac{D_{p}}{2}\right)}^{3}\rho_{p}N\left(D_{p}\right) \end{array}$$

$\rho_{p}$ is the material density of the PM, which can be considered the same as the material density of the e-liquid ($\rho_{p}$ = 1.12 g/mL). The differential mass concentration per logarithmic interval of $D_{p}$, given as $\frac{dC}{dlogD_{p}}$, is plotted in Figure 3f. The peak diameter of $C(D_{p})$ and that of $N(D_{p})$ are quite different because the mass of a particle scales as the third power of the diameter. Our SMPS measurement results of e-cigarette aerosols are consistent with those reported in the literature [26,28,29,63].

From the PM mass concentration and the volume of the dilution box, given as $V_{box}$ = 94.6 L, the size-binned total PM mass in the three bins are calculated by Equation (12). The results are shown in Figure 3g. The PM mass in each size bin is typically in the mg range and is significantly increasing from $M_{1}$ (smaller PM) to $M_{3}$ (larger PM).

(12) $$\begin{array}{c} M_{1}=\int_{100\;nm}^{300\;nm}\left(\frac{dC}{dlogD_{p}}\right)V_{box}dlogD_{p}\\ M_{2}=\int_{300\;nm}^{600\;nm}\left(\frac{dC}{dlogD_{p}}\right)V_{box}dlogD_{p}\\ M_{3}=\int_{600\;nm}^{1000\;nm}\left(\frac{dC}{dlogD_{p}}\right)V_{box}dlogD_{p} \end{array}$$

### 3.6. Training, Validation, and Testing of the Neural Network

**Figure 4:**
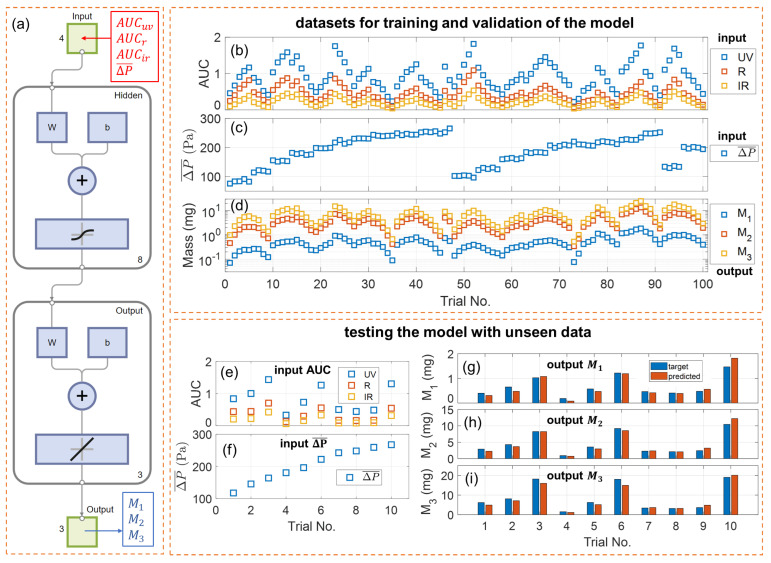
Training, validation, and testing of the neural network. (**a**) Schematic of the two-layer feedforward neural network model. (**b**) Integrals of optical attenuation, (**c**) mean inhalation pressure, and (**d**) measured size-binned PM mass collected from the 100 puffs with varying e-cigarette puffing conditions for training and validation of the model. (**e**) Integrals of optical attenuation and (**f**) mean inhalation pressure collected from the 10 additional puffs for testing the model with unseen data. Comparison of the PM mass by reference measurement (target) and the PM mass calculated by the model (predicted) for (**g**) size bin #1 (100–300 nm), (**h**) size bin #2 (300–600 nm), and (**i**) size bin #3 (600–1000 nm).

A schematic of a default neural network model used in this work is given in Figure 4a. It is essentially a shallow two-layer feedforward neural network with a sigmoid transfer function in the hidden layer and a linear transfer function in the output layer. The layer size, defined as the number of neurons in the hidden layer, is eight by default. Each input (predictor) is a 4 × 1 column vector [$AUC_{uv}$; $AUC_{r}$; $AUC_{ir}$; $\bar{\Delta P}$] and each output (response) is a 3 × 1 column vector [$M_{1}$; $M_{2}$; $M_{3}$]. In the hidden layer, the size of the weight matrix “W” is 8 × 4, and that of the bias vector “b” is 8 × 1. In the output layer, the size of the weight matrix is 3 × 8, and that of the bias vector is 3 × 1. The neural network model has a total of 67 parameters, which makes it a very lightweight model, ideal for deployment on low-power microcontrollers in portable devices like e-cigarettes.

The training, validation, and testing of the neural network model were carried out using the Neural Net Fitting toolbox of MATLAB. The computer used in this work is a ThinkPad P1 Gen 5 laptop, equipped with a CPU (12th Gen Core i7-12700H processor 3.5 GHz, manufactured by Intel, Santa Clara, CA, USA) and a GPU (RTX A2000, manufactured by Nvidia, Santa Clara, CA, USA). For training and validation of the neural network model, 100 pairs of input/output data were attained from 100 different puffs using diversified puffing conditions as listed in Table S1. Out of these data, 85% were selected for training the model and the remaining 15% for validation, which aids the training process to determine the optimal model parameters. The selection and assignment of data were random and were automatically determined by the toolbox. The training algorithm used in this work was “Levenberg–Marquardt” backpropagation for its high performance and robustness. For testing the neural network with unseen data, an additional 10 pairs of input/output data were acquired from 10 new puffs and used on the neural network model to evaluate its performance.

The performances for training, validation, and testing of the neural network are evaluated by the mean squared error (MSE) for the PM mass in three size bins, given in Equation (13),

(13) $$\begin{array}{c} MSE_{1}=\frac{1}{n}\sum_{k=1}^{n}{\left(M_{1,k}-{\hat{M}}_{1,k}\right)}^{2}\\ MSE_{2}=\frac{1}{n}\sum_{k=1}^{n}{\left(M_{2,k}-{\hat{M}}_{2,k}\right)}^{2}\\ MSE_{3}=\frac{1}{n}\sum_{k=1}^{n}{\left(M_{3,k}-{\hat{M}}_{3,k}\right)}^{2} \end{array}$$

in which ${\hat{M}}_{1,k}$, ${\hat{M}}_{2,k}$, ${\hat{M}}_{3,k}$ are the predicted values by the neural network model while $M_{1,k}$, $M_{2,k}$, $M_{3,k}$ are the target values (ground truth from reference measurements) for puff No. *k*. The performance for training and validation is calculated from all 100 data used for training and validation. The performance for testing is calculated from the 10 additional data collected for testing. It should be noted that the calculated MSE has a unit of mg^2^.

To evaluate the accuracy of the multi-spectral sensor, in terms of quantification of size-binned PM mass, the mean relative error (MRE) is also calculated for the three separate size bins, according to Equation (14).

(14) $$\begin{array}{c} MRE_{1}=\frac{1}{n}\sum_{k=1}^{n}\left|\frac{M_{1,k}-{\hat{M}}_{1,k}}{M_{1,k}}\right|\\ MRE_{2}=\frac{1}{n}\sum_{k=1}^{n}\left|\frac{M_{2,k}-{\hat{M}}_{2,k}}{M_{2,k}}\right|\\ MRE_{3}=\frac{1}{n}\sum_{k=1}^{n}\left|\frac{M_{3,k}-{\hat{M}}_{3,k}}{M_{3,k}}\right| \end{array}$$

To assess if the model is overpredicting or underpredicting, the mean bias error (MBE) is calculated according to Equation (15).

(15) $$\begin{array}{c} MBE_{1}=\frac{1}{n}\sum_{k=1}^{n}\left(M_{1,k}-{\hat{M}}_{1,k}\right)\\ MBE_{2}=\frac{1}{n}\sum_{k=1}^{n}\left(M_{2,k}-{\hat{M}}_{2,k}\right)\\ MBE_{3}=\frac{1}{n}\sum_{k=1}^{n}\left(M_{3,k}-{\hat{M}}_{3,k}\right) \end{array}$$

## 4. Results and Discussion

The dataset of 100 puffs collected for training and validation of the model is plotted in Figure 4b–d. The data appeared in an oscillating pattern because the puffing conditions for the 100 trials were implemented by periodically varying inhalation pressure, button pusher duration, and atomizer power, as listed in Table S1. Each individual parameter varied in its own periodic fashion within its own given range. The combined effects of these parameters resulted in periodically varying aerosol output and, thereby, sensor signals. The dataset of 10 new puffs collected for testing the model are plotted in Figure 4e–i.

### 4.1. Performance of the Default Neural Network Model

The performance of the default neural network model, as well as other models trained with various configurations, are listed in Table 1. The default model, “NN01”, was trained with the complete set of input ($AUC_{uv}$, $AUC_{r}$, $AUC_{ir}$, $\bar{\Delta P}$) and a layer size of 8. The training was completed with 8 epochs. The MSE for training and validation is 0.009939, 0.2416, and 0.5393 for $M_{1}$, $M_{2}$, and $M_{3}$, respectively. A significantly increasing MSE for $M_{3}$ is a direct consequence of a larger $M_{3}$ measured from the e-cigarette aerosols. The performance for testing the model with unseen data from 10 new puffs, i.e., the MSE calculated for $M_{1}$, $M_{2}$, and $M_{3}$, is 0.01935, 0.5296, and 2.1065, respectively. The MSE for testing is a little higher than that for training and validation because the dataset for testing was unseen by the model.

Figure 4g–i compare the target values (ground truth) and the model-predicted values for PM mass in the three size bins. For the wide range of e-cigarette puffing conditions, these two values match closely, and the calculated mean relative error (MRE) is 19.0% for $M_{1}$, 13.5% for $M_{2}$, and 14.9% for $M_{3}$. These values validate the performance of our multi-spectral sensor in quantifying the size-binned PM mass, with an accuracy of about 81.0% for a PM of 100–300 nm, 86.5% for a PM of 300–600 nm, and 85.1% for a PM of 600–1000 nm. Given the sensor’s simple construction and the direct collection of signals from high-concentration, undiluted e-cigarette aerosols, this level of accuracy is notably significant and sufficiently reliable for point-of-interest characterization of vaping aerosols. Even particles smaller than 300 nm can be instantly quantified with a decent accuracy, which usually requires sophisticated bulky aerosol instruments.

The calculated mean bias error (MBE) is 0.00935 mg for $M_{1}$, 0.00921 mg for $M_{2}$, and 0.653 mg for $M_{3}$. The mean PM mass from the 10 testing puffs is 0.688 mg for $M_{1}$, 4.643 mg for $M_{2}$, and 8.765 mg for $M_{3}$. All MBE values are positive, suggesting the overall residues between actual values and the predicted values are positive. The ratio between MBE and mean PM mass is about 1.36%, 0.20%, and 7.44% for $M_{1}$, $M_{2}$, and $M_{3}$, respectively. Based on these calculations, the model does not overpredict or underpredict $M_{1}$ or $M_{2}$, as the relevant ratios are insignificant; however, it slightly underpredicts $M_{3}$. This trend may be linked with the low contrast of wavelength-dependent scattering for PM in size bin #3 (as shown in Figure 1b), and the approximation by neglecting PM above 1000 nm. It will be investigated in our future work.

We evaluated the response time of the default neural network using double-precision floating point numbers as model parameters. On the ThinkPad laptop used for training, it took about 18.90 microseconds to output one result. In the future work, to accomplish in-device sensing of electronic cigarette aerosols, the neural network model will be incorporated into an MCU inside the device. We tested the same neural network model on a popular MCU, ESPRESSIF ESP32 WROOM (dual core, 240 MHz), and it took about 116.80 microseconds to output one result. The short response time confirmed that the neural network is computationally simple, and it can instantly report the size-binned PM mass.

### 4.2. Puff Topography with Size-Binned PM Mass

The multi-spectral sensor can open new avenues for monitoring size-binned PM mass with aerosol sensors built inside e-cigarette devices. The prototype sensor box shown in Figure 2a is a compact, portable device but still too bulky for in-device integration; however, there is a huge potential to scale down its form factor into a compact chip-size sensor, owing to the simple optical configuration of the sensor. Compared with our previous work, which is limited to quantifying the entire PM only [40]; this new sensor can greatly extend the capabilities of tracking a user’s puff topography with further size-specific information of PM.

**Figure 5:**
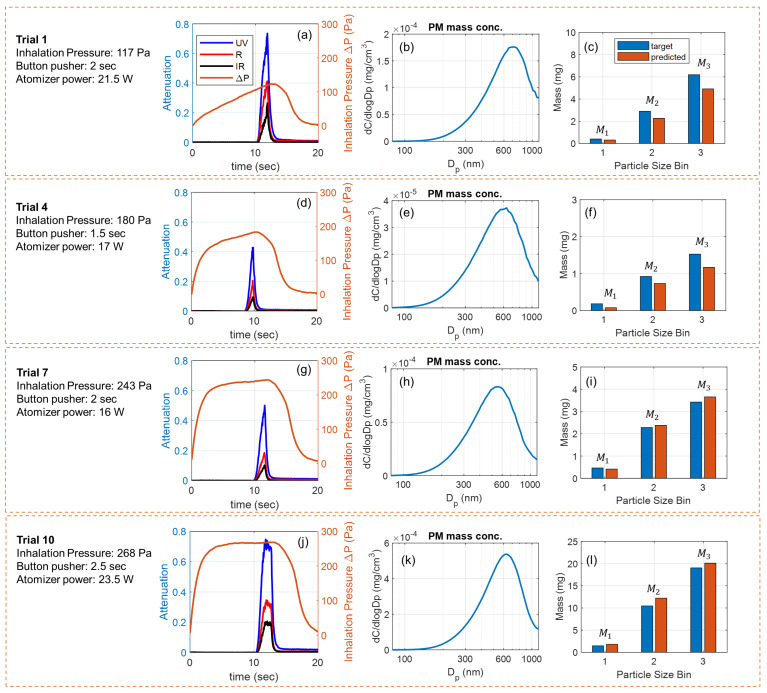
Experimental results of four exemplary puffs used for testing the model. (**a**) Sensor signals, (**b**) measured PM mass concentration of the diluted aerosol, and (**c**) the target and model-predicted PM mass for the three size bins from puff No. 1. (**d**–**f**) Results from puff No. 4. (**g**–**i**) Results from puff No. 7. (**j**–**l**) Results from puff No. 10.

Figure 5 shows four exemplary puffs collected for testing the model. The puffing conditions, sensor signals, reference measurement results, and size-binned PM mass from each puff are provided in parallel. Given varying puffing conditions (inhalation pressure, button pusher duration, and atomizer power), the physical properties of generated e-cigarette aerosols, in terms of peak concentration and peak diameter, shift from puff to puff. Typically, a higher atomizer power generates an aerosol with higher concentration and a larger peak diameter [31,63]. A longer button pusher duration and a larger inhalation pressure (e.g., higher aerosol flow rate) generate aerosols with more total PM [38]. Using our data-driven multi-spectral sensor, the size-specific information about the PM mass in the puff can be tracked with reliable accuracy from puff to puff. Since the deposition of PM in lungs strongly depends on the particle size distribution [53,54,55,56], our sensor can potentially measure and track lung deposition of e-cigarette aerosols for the users to support personalized health and wellness, which will be investigated in our future work.

### 4.3. Configuration of the Neural Network Models

We studied how the model’s configuration parameters affect the performance of the neural network, as summarized in Table 1. The default model “NN01” delivers the best overall performance. It may be argued that since e-cigarette aerosols from the same e-cigarette and e-liquid can have roughly similar particle size distribution profiles, the size-binned PM mass may be merely a function of the mass of total PM, which can be quantified using a single wavelength or two wavelengths. To test this hypothesis and validate the roles of all three wavelengths, models “NN02”–“NN09” were deployed by training the neural network with optical signals from only one or two wavelengths. For example, model “NN02” was trained with $\bar{\Delta P}$ and $AUC_{ir}$ (one wavelength) and model “NN05” with $\bar{\Delta P}$, $AUC_{uv}$, and $AUC_{ir}$ (two wavelengths). Two different layer sizes were applied to account for the effects of the number of input predictors on the neural network. By comparing the results, models “NN02”–“NN09” all had higher MSEs than the default model for PM mass in all three size bins, especially for the unseen dataset used for testing. This trend suggests that more wavelengths can significantly improve the performance of the sensor by better resolving the PM of different size ranges. This observation is also consistent with the Mie scattering spectra shown in Figure 1b, as more wavelengths can provide more information to differentiate particles of different sizes.

In addition, we investigated how the layer size affects the neural network using model “NN10”, which implements more neurons (layer size 12) in the hidden layer than the default model (layer size 8). By increasing the layer size, the performance for training and validation is improved, as MSE for all three size bins became smaller, which indicates that the neural network with more parameters can fit the training and validation dataset better. However, the performance for testing with unseen data declines as MSE for $M_{2}$ and $M_{3}$ significantly increase. This trend in layer size suggests that model “NN10” has overfit.

### 4.4. Particle Size Bins

In this work, three size bins for PM mass were defined as size bin #1 (100–300 nm), size bin #2 (300–600 nm), and size bin #3 (600–1000 nm). These size bins were decided based on the following considerations. Firstly, the PM of e-cigarette aerosols are mainly fine particles and ultrafine particles. Moreover, the PM that can reach the tracheobronchial and pulmonary region of the respiratory tract are mostly particles smaller than 1000 nm [53,54,55,56]. Therefore, size bins covering particles up to 1000 nm can effectively quantify the PM mass in e-cigarette aerosols to monitor the potential health risks to users. Secondly, for ultrafine particles, which are smaller than 100 nm, the optical attenuation signals are too weak, compared to other particles in the size range [100 nm, 1000 nm]. In principle, it would be unpractical to resolve ultrafine particles using the optical configuration in this work. In-device quantification of ultrafine particles can potentially be achieved using other emerging compact aerosol-sensing platforms, such as nanoplasmonic sensors [64,65,66] and MEMS-based sensors [67,68]. In addition, this work focuses on quantifying the mass of PM, as the mass of ultrafine particles is negligible in comparison to that of particles in the size range [100 nm, 1000 nm]. It should be mentioned that ultrafine particles are typically characterized by their number concentration, rather than their mass concentration to better assess their potential health risks [69,70]. Therefore, we implemented size bins inside the range [100 nm, 1000 nm] to quantify the PM mass using our multi-spectral sensor. Thirdly, our multi-spectral sensor relies on the contrast in spectral responses of three wavelengths to differentiate PM in different size bins. As shown in Figure 1, the scattering spectrum for each wavelength has a transition point, which separates a gentle slope (for larger particles) from a steep slope (for smaller particles). These transition points from three spectra, at 300 nm, 600 nm, and 1000 nm, are used as the cut-off diameters of three size bins. The overall spectral responses of three wavelengths provide good contrast to differentiate PM in these three size bins. In our future work, we will further investigate the optimal settings of wavelengths and size bins for multi-spectral sensors.

### 4.5. Performance on Unseen E-Cigarettes or E-Liquids

The training, validation, and testing of the neural network presented above are based on the default e-cigarette (Nautilus PrimeX) loaded with the default e-liquid (KSPR). The performance of the default neural network “NN01” for unseen e-cigarettes or e-liquids is important for understanding the versatility of the sensor. As listed in Table 2, different combinations of e-cigarettes and e-liquids have been tested. For each configuration, 10 puffs were collected from the device connected to the sensor box. Configurations 01, 02, and 03 compare the performance on the same default e-cigarette but different e-liquids. With e-liquid ENVY or DewBerry, the MSE for $M_{1}$, $M_{2}$, $M_{3}$, all increased significantly. We attribute this trend to the optical properties of e-liquids. As shown in Figure S2, e-liquid ENVY appears brown and more opaque than KSPR, while DewBerry appears colorless and more transparent than KSPR. Optical transmission measurements, as shown in Figure S2c,d, further reveal that three e-liquids have distinctive optical absorption peaks and amplitudes. Since our sensor relies on optical attenuation signals from particles in e-cigarette aerosols, the optical absorption of the particle material will affect the sensor signals. Consequently, aerosols generated from the unseen e-liquids cause the neural network to make predictions that noticeably deviate from the target values.

Configurations 01, 04, and 05 compare the performance of the neural network on different e-cigarettes with the same default e-liquid. Using a different e-cigarette, SUORIN or SMOK S-PRIV, the MSE increased significantly. Particularly, the MSE for SMOK S-PRIV increased by more than 10 times. The main differences in the three e-cigarettes are their flow characteristics, which play a significant role in determining the aerosol flow rate and the size-binned mass, as described in Equations (3) and (4). The flow characteristics of three e-cigarettes have been characterized in pressure testing using a commercial vaping machine, as shown in Figure S1c. A higher pressure reading indicates a larger flow resistance of the device. As listed in Table 2, the trend of MSE for three e-cigarettes is consistent with the trend of their flow resistance. Since the SMOK S-PRIV device has a very different flow resistance than the default e-cigarette, the neural network trained for the default e-cigarette cannot predict accurate values for SMOK S-PRIV. The neural network can perform moderately better for SOURIN because its flow resistance is closer to that of the default device.

These results on unseen e-cigarettes or e-liquids indicate that our data-driven multi-spectral sensor in this work can only give high-accuracy output for the default e-cigarette and default e-liquid. However, this limitation is due to a simple shallow neural network trained with a default device and e-liquid only. In our future work, we will implement more advanced machine learning algorithms to train deep neural networks with a wide range of e-cigarettes and e-liquids. Additional input predictors will be added to the neural network to account for variations in e-cigarettes and e-liquids.

### 4.6. Machine Learning

The neural network model employed in this study is highly lightweight, with only 67 parameters, making it ideal for embedding into the low-power microprocessors of e-cigarettes. A few approximations have been made to the wavelength-specific optical attenuation from aerosols, in order to simplify the data preparation algorithm and the neural network model. There is still significant room for improvement in both the algorithm and the neural network, which will be explored in our future work.

Machine learning has been used for behavioral studies related to vaping, such as predicting e-cigarette usage and nicotine addiction [71,72]. Our data-driven multi-spectral sensor, as the first demonstration of using machine learning to directly characterize high-concentration e-cigarette aerosols, is positioned to become a powerful digital tool for managing the health risks of vaping. Tracking size-binned PM mass over long-term e-cigarette usage will provide valuable quantitative data for studying vaping behaviors, potentially enabling new approaches for vaping reduction or vaping cessation.

### 4.7. Limitations and Future Work

The work presented in this article focuses on validating the proposed sensing mechanism based on multi-spectral optical sensing and machine learning. There are limitations to the present work and room for improvement, which will be the focus of our future work. The sensor box, as a prototype, served its purpose for proof of concept as a portable sensor device. However, it is relatively too large for in-device e-cigarette aerosol monitoring. In our future work, integrated optoelectronic components capable of multi-spectral sensing will be used to build compact sensors on a sub-centimeter scale. In this work, a dataset of 100 trials was used to train the neural network for one device configuration. In our future work, much more comprehensive datasets covering more device configurations and more variables, including atomizer power, e-liquid compositions, and flow resistance, will be collected to train a neural network that is versatile for a much broader range of device configurations and puffing conditions. In this work, the three wavelengths and three size bins were determined based on typical e-cigarette aerosol particle size distribution profiles and the optical contrast in scattering efficiency. In our future work, more wavelengths will be implemented to probe optical responses from the e-cigarette aerosol, and optimal size bins sorted into more channels will be studied.

### 4.8. Other Applications

The sensor in this work was developed for sensing e-cigarette aerosols but it can also be used for other aerosol sensing applications, after necessary adaptions. For example, it can be used with inhalation drug delivery devices to monitor the dose of aerosolized drugs inhaled by the patient. The multi-spectral sensing combined with machine learning can be implemented to measure size-binned PM concentrations in the environment, for both indoor and outdoor air quality monitoring. Specifically, it can be used to monitor environmental hazards including severe air pollution caused by wildfires.

### 4.9. Comparison with Other Optical Approaches

Exiting optical approaches for measuring aerosols, especially e-cigarette aerosols, typically quantify aerosol concentration [41,42,47], particle size distribution [44,45,46], mass median diameter [32], and volatility [43]. Our sensor measures the size-binned PM mass, determined by both particle size distribution and mass flow rate. The sensor output can be directly used for predicting the lung deposition of e-cigarette aerosols. Furthermore, existing works measure aerosols at low or medium concentrations, such as diluted e-cigarette aerosols [43,45,50,51,52]. In comparison, our sensor can directly characterize high-concentration e-cigarette aerosols with a decent accuracy.

## 5. Conclusions

We introduced a portable aerosol sensor based on multi-spectral optical sensing and machine learning for measuring e-cigarette aerosols at points of interest. Using three wavelengths of light at 370 nm, 640 nm, and 940 nm configured into optical transmission mode, high-concentration undiluted e-cigarette aerosols were directly characterized for size-binned PM mass. The working principle is based on the contrast of wavelength-specific optical attenuation to different particle sizes. The neural network was trained with a dataset for 100 puffs collected from a wide range of puffing conditions on the default e-cigarette and e-liquid. The achieved accuracy in quantifying the PM mass reached 81.0% for a PM of 100–300 nm, 86.5% for 300–600 nm, and 85.1% for 600–1000 nm for detecting e-cigarette aerosols from the default e-cigarette and e-liquid. The performance of the neural network declined when tested with new e-cigarettes or e-liquids due to changes in flow characteristics of e-cigarettes and optical properties of e-liquids. Various configurations of neural network models have been studied to find the optimal model parameters. The crucial roles of the three wavelengths were confirmed by comparing the default model with models trained using only one or two wavelengths. The sensor’s applications in monitoring users’ puff topography with estimation of lung deposition of e-cigarette aerosols were also discussed.

## Figures and Tables

**Table 1:** Performance of neural network models trained with various configurations and parameters. Datasets for training, validation, and testing were collected from the Nautilus PrimeX e-cigarette loaded with KSPR e-liquid.

Model Ref. No.	Input	Layer Size	Epoch	Performance for Model Training and Validation			Performance for Testing the Model with Unseen Data		
				MSE for *M1*	MSE for *M2*	MSE for *M3*	MSE for *M1*	MSE for *M2*	MSE for *M3*
**NN01 (Default)**	**$\bar{\Delta P}$ and AUC for UV, Red & IR**	**8**	**8**	**0.009939**	**0.2416**	**0.5393**	**0.01935**	**0.5296**	**2.1065**
NN02	$\bar{\Delta P}$ and AUC for IR only	8	4	0.03886	1.1561	2.5582	0.05941	2.4227	9.7990
NN03	$\bar{\Delta P}$ and AUC for Red only	8	9	0.05745	1.8651	4.7808	0.06869	2.5469	11.3641
NN04	$\bar{\Delta P}$ and AUC for UV only	8	4	0.03943	1.4460	3.7362	0.05638	2.3871	9.4459
NN05	$\bar{\Delta P}$ and AUC for UV & IR	8	5	0.03525	1.0848	2.0966	0.04068	2.0939	6.9357
NN06	$\bar{\Delta P}$ and AUC for IR only	5	57	0.02878	0.9960	2.0654	0.04487	2.0051	7.1812
NN07	$\bar{\Delta P}$ and AUC for Red only	5	10	0.04971	1.8346	4.8166	0.06208	3.2874	14.0912
NN08	$\bar{\Delta P}$ and AUC for UV only	5	24	0.03350	1.0728	2.4257	0.05101	2.4485	12.1907
NN09	$\bar{\Delta P}$ and AUC for UV & IR	5	7	0.03948	1.2161	2.6419	0.03799	1.1729	5.0783
NN10	$\bar{\Delta P}$ and AUC for UV, Red & IR	12	17	0.008897	0.1321	0.3017	0.01626	1.6694	5.4919

**Table 2:** Testing the neural network model “**NN01**” for different e-liquids and e-cigarette devices.

Config. No.	Electronic Cigarette Device	E-Liquid	Performance for Testing the Model with Unseen Data			E-Liquid Color	* Device Pressure Testing (Pa)	Notes
			MSE for *M1*	MSE for *M2*	MSE for *M3*			
**01**	**Nautilus PrimeX**	**KSPR**	**0.01935**	**0.5296**	**2.1065**	**light yellow**	**898.5**	**Default configuration**
02	Nautilus PrimeX	ENVY	0.1042	4.1486	8.5527	brown	898.5	E-liquid more opaque than KSPR
03	Nautilus PrimeX	DewBerry	0.1150	3.7665	5.4013	clear	898.5	E-liquid more transparent than KSPR
04	SUORIN	KSPR	0.07011	2.1045	5.2330	light yellow	1601.0	The e-cigarette has higher flow resistance than default
05	SMOK S-PRIV	KSPR	0.2673	11.5140	25.7882	light yellow	2518.3	The e-cigarette has the highest flow resistance

* Pressure testing of all three e-cigarette devices was carried out using a Hokord HAVC vaping machine. A higher pressure indicates a device with larger flow resistance.

## Data Availability

The original contributions presented in this study are included in the Supplementary Material. Further inquiries can be directed to the corresponding author.

## Supplementary Materials

The following supporting information can be downloaded at: https://www.mdpi.com/article/10.3390/s24217082/s1, Figure S1: Electronic cigarettes and the experimental setup for pressure testing. Figure S2: Optical properties of e-liquids. Figure S3: Experimental setup for collecting sensor data and conducting reference PM measurements of the generated e-cigarette aerosols. Table S1: Experimental conditions for collecting a dataset of 100 puffs for training and validation. Table S2: Experimental conditions for collecting a dataset of 10 new puffs for testing the model. Original data used in training, validating, and testing the neural network.

## Abbreviations

The following abbreviations are used in this article:

E-Cigarette	Electronic Cigarette
PM	Particulate Matter
UV	Ultraviolet
R	Red
IR	Infrared
FWHM	Full Width at Half Maximum
MCU	Micro-Controller Unit
ADC	Analog-to-Digital Conversion
TIA	Transimpedance Amplifier
LED	Light Emitting Diode
MSE	Mean Squared Error
MRE	Mean Relative Error
MBE	Mean Bias Error
NN	Neural Network
SMPS	Scanning Mobility Particle Sizer
CPC	Condensation Particle Sizer
EC	Electrostatic Classifier
DMA	Differential Mobility Analyzer
List of Symbols	
$I_{uv}\left(t\right)$, $I_{r}\left(t\right)$, and $I_{ir}\left(t\right)$	Optical intensity signal for UV, Red, and IR wavelength, respectively
$A_{uv}\left(t\right)$, $A_{r}\left(t\right)$, and $A_{ir}\left(t\right)$	Optical attenuation in response to an e-cigarette aerosol flow for UV, Red, and IR wavelength, respectively
$AUC_{uv}$, $AUC_{r}$, and $AUC_{ir}$	Integrated optical attenuation (area under the curve) of $A_{uv}\left(t\right)$, $A_{r}\left(t\right)$, and $A_{ir}\left(t\right)$, respectively
ΔP(t)	Inhalation pressure measured by the pressure sensor
$c_{1}\left(t\right)$, $c_{2}\left(t\right)$, and $c_{3}\left(t\right)$	Mass concentration of PM in size bin 1, 2, and 3, respectively, as a function of time for the aerosol flow
Q(t)	Volumetric flow rate of the e-cigarette aerosol, as a function of time
$M_{1}$, $M_{2}$, and $M_{3}$	Mass of PM in size bin 1, 2, and 3, respectively

$D_{p}$	Particle diameter
$N(D_{p})$	PM number concentration of the diluted aerosol in the dilution box, as a function of particle diameter
$C(D_{p})$	PM mass concentration of the diluted aerosol in the dilution box, as a function of particle diameter
$V_{box}$	Volume of the dilution box
