# Supplementary material for: Quantification of Size-Binned Particulate Matter in Electronic Cigarette Aerosols Using Multi-Spectral Optical Sensing and Machine Learning
Source: Sensors (Basel). 2024 Nov 3;24(21):7082. doi: 10.3390/s24217082 (PMC11548654; doi:10.3390/s24217082)
Supplement: Supplementary file 1 [file sensors-24-07082-s001.zip › Supplementary_Information_E_Cigarette_Aerosol_Multispectral_Sensing.pdf]

Quantification of Size-Binned Particulate Matter in Electronic Cigarette Aerosols Using Multi-Spectral Optical Sensing and Machine Learning

(supplementary information)

Hao Jiang [1,†*] , and Keith Kolaczyk [1,†]

[1] Department of Biomedical Engineering, Lawrence Technological University, 21000 W 10 Mile Road, Southfield, Michigan 48075, USA; hjiang@ltu.edu; kkolaczyk@ltu.edu
* Correspondence: hjiang@ltu.edu
[†] These authors contributed equally to this work.

<u>List of supplementary information:</u>

Figure S1: Electronic cigarettes and the experimental setup for pressure testing.

Figure S2: Optical properties of e-liquids.

Figure S3: Experimental setup for collecting sensor data and conducting reference PM measurements of the generated e-cigarette aerosols.

Table S1: Experimental conditions for collecting a dataset of 100 puffs for training and validation.

Table S2: Experimental conditions for collecting a dataset of 10 new puffs for testing the model.

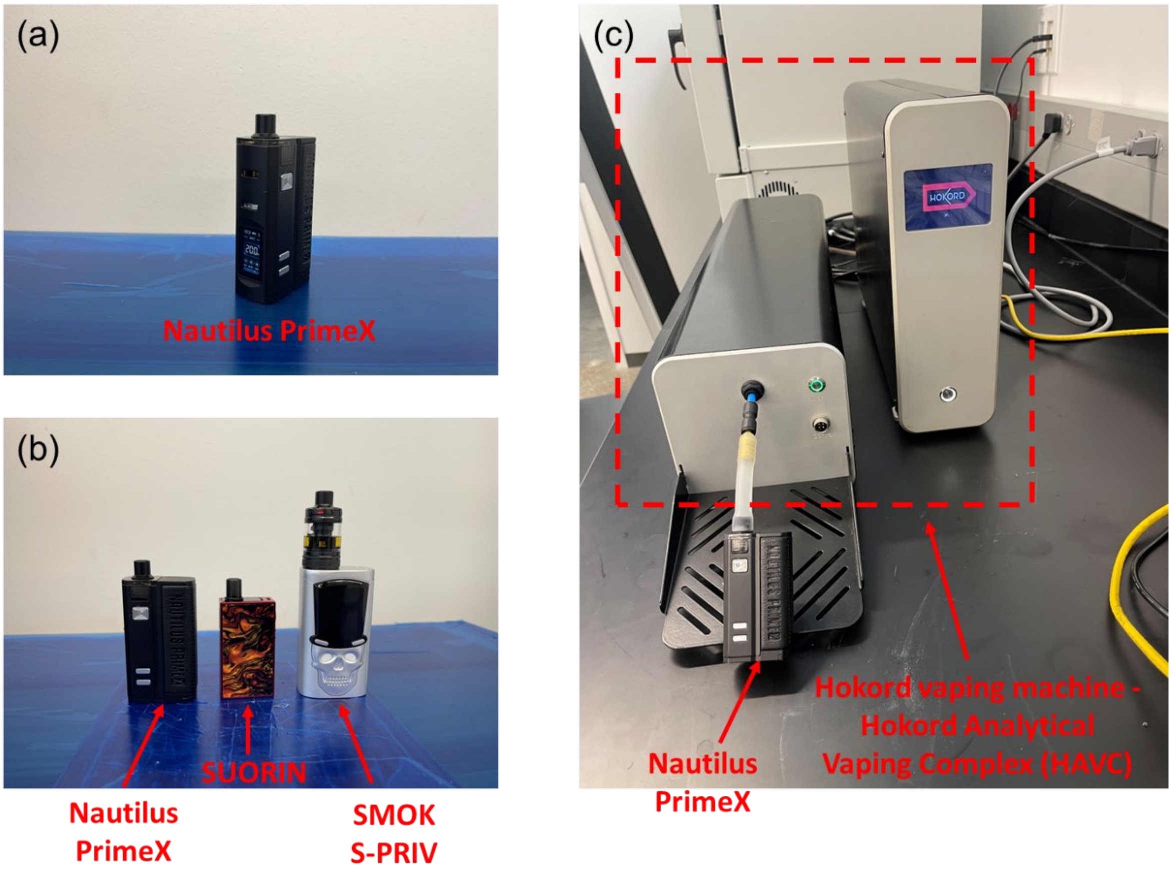

Figure S1: Electronic cigarettes and the experimental setup for pressure testing. (a) Photograph of Nautilus PrimeX, the default device used in this work. (b) Photograph of all three electronic cigarette devices used in this work: Nautilus PrimeX, SUORIN ELITE, and SMOK S-PRIV. (c) Experimental setup for pressure testing (flow resistance testing) of the electronic cigarette devices using a Hokord Analytical Vaping Complex (HAVC) vaping machine.

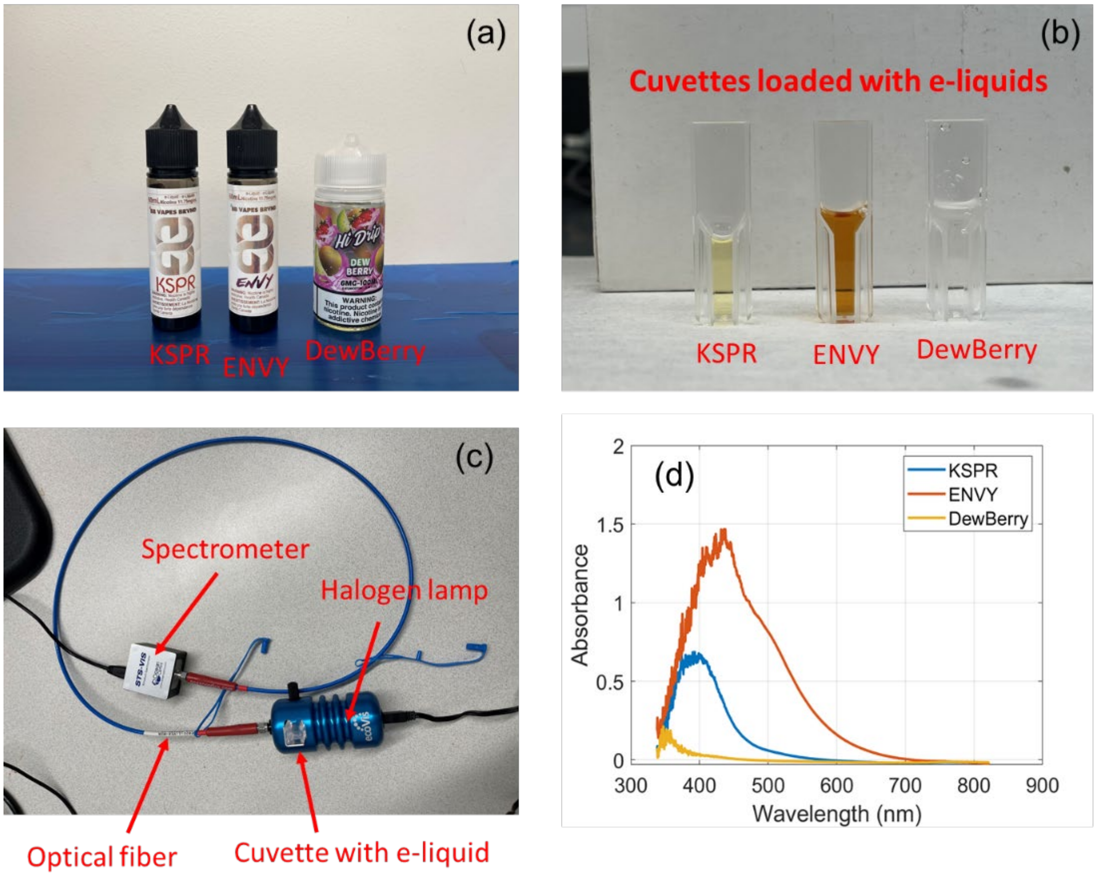

Figure S2: Optical properties of e-liquids. (a) Photographs of three e-liquids used in this work in original bottles: KSPR, ENVY, and DewBerry. (b) E-liquids loaded in cuvettes for optical measurements. The path length of the cuvette (for light transmission) is 10 mm. KSPR appears light yellow in color; ENVY appears brown; DewBerry appears clear. (c) Optical setup to measure the absorbance spectra of the e-liquids. (d) Measured absorbance spectra of three e-liquids. Absorption peak wavelength is 392 nm for KSPR, 434 nm for ENVY, and 351 nm for DewBerry.

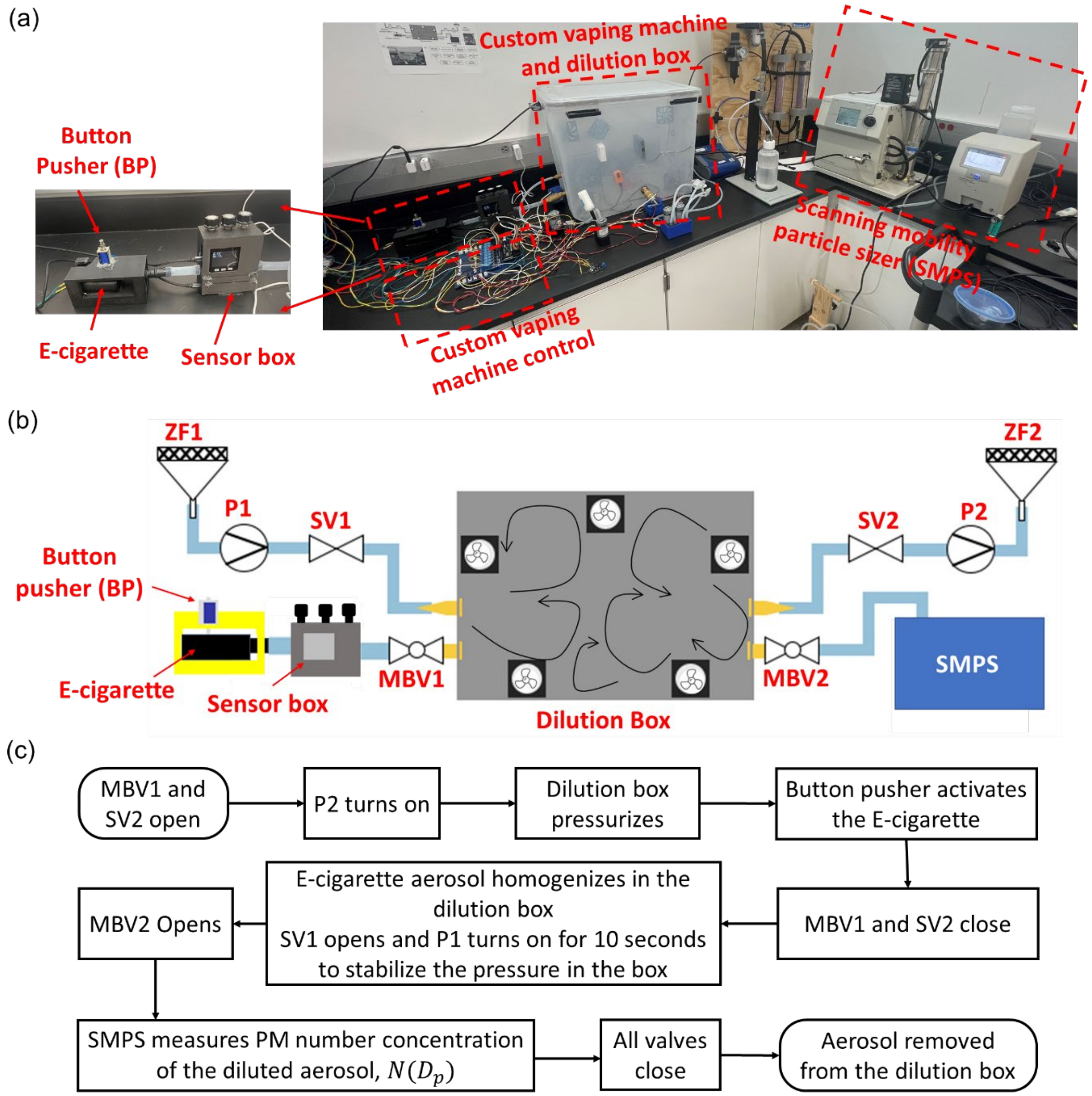

Figure S3: Experimental setup for collecting sensor data and conducting reference PM measurements of the generated e-cigarette aerosols. (a) Photograph of the experimental setup. (b) Schematic of the experimental setup. (c) Flowchart of the system's functionality for measuring PM in one puff.

Table S1: Experimental conditions for collecting a data set of 100 puffs for training and validation. 100 puffs were activated from the Nautilus PrimeX e-cigarette (the default e-cigarette device) with KSPR e-liquid (the default e-liquid). The inhalation pressure, button pusher duration, and atomizer power were diversified to generate a dataset representing a wide range of puffing conditions. The inhalation pressure is controlled through the pressurization time used in the dilution box of the custom vaping machine. A longer pressurization time gives a higher inhalation pressure.

Trial (puff) No.	Custom vaping machine dilution box pressurization time (sec)	Button pusher duration (sec)	Atomizer power (watt)
1	7	2	15
2	7	2	17
3	7	2	20
4	7	2	22
5	7	2	25
6	12	1.5	24
7	12	1.5	21
8	12	1.5	18
9	12	1.5	16
10	17	2.5	17
11	17	2.5	19
12	17	2.5	21
13	17	2.5	23
14	22	2.25	22
15	22	2.25	25
16	22	2.25	20
17	22	2.25	17
18	22	2.25	15
19	28	1.75	16.5
20	28	1.75	18.5
21	28	1.75	20.5
22	29	1.75	23.5
23	34	3	21
24	34	3	19
25	34	3	17
26	34	3	15
27	40	2	17
28	40	2	19.5
29	40	2	21.5
30	40	2	24
31	48	1.5	25
32	48	1.5	24
33	48	1.5	21
34	48	1.5	17
35	48	1.5	15
36	56	2.25	16
37	56	2.25	18
38	56	2.25	20

39	56	2.25	21
40	56	2.25	23
41	66	1.75	23.5
42	66	1.75	21.5
43	66	1.75	19.5
44	66	1.75	17.5
45	66	1.75	15.5
46	80	3	17
47	150	2	20
48	9	2.5	15
49	9	2.5	17
50	9	2.5	20
51	9	2.5	22
52	9	2.5	25
53	15	2	24
54	15	2	21
55	15	2	18
56	15	2	16.5
57	15	2	15.5
58	20	1.75	16
59	20	1.75	18.5
60	20	1.75	20.5
61	20	1.75	22.5
62	20	1.75	25
63	25	2.5	15.5
64	25	2.5	17
65	25	2.5	19
66	25	2.5	21
67	25	2.5	23
68	31	2.25	24
69	31	2.25	21.5
70	31	2.25	19.5
71	31	2.25	17.5
72	31	2.25	16.5
73	37	1.5	15
74	37	1.5	18
75	37	1.5	22
76	37	1.5	23
77	37	1.5	25
78	44	2	24.5
79	44	2	22.5
80	44	2	19
81	44	2	16.5
82	44	2	15
83	52	3	16
84	52	3	17
85	52	3	19
86	52	3	21
87	52	3	24

88	150	2	24.5
89	150	2	22.5
90	150	2	17.5
91	150	2	15
92	13	3	16
93	13	3	18
94	13	3	22
95	13	3	20
96	30	2	25
97	30	2	22.5
98	30	2	20
99	30	2	17.5
100	30	2	15

Table S2: Experimental conditions for collecting a dataset of 10 new puffs for testing the model. 10 puffs were activated from the Nautilus PrimeX e-cigarette (the default e-cigarette device) with KSPR e-liquid (the default e-liquid). The inhalation pressure, button pusher duration, and atomizer power were diversified to generate a dataset representing a wide range of puffing conditions. The combinations of the condition parameters have not been used in Table S1 to ensure the generated data are truly unseen by the model.

Trial (puff) No.	Custom vaping machine dilution box pressurization time (sec)	Button pusher duration (sec)	Atomizer power (watt)
1	11	2	21.5
2	15	3	15
3	19	2.5	24
4	23	1.5	17
5	27	2	19
6	38	2.25	25
7	50	2	16
8	60	1.5	20
9	80	1.75	18.5
10	120	2.5	23.5
